# Death receptor-induced apoptosis signalling - essential guardian against autoimmune disease

**DOI:** 10.1186/ar3563

**Published:** 2012-02-09

**Authors:** Andreas Strasser, Lorraine A O'Reilly, Philipp Jost, Thomas Kaufmann, Stephanie Grabow, Elizabeth Kruse, Lin Tai, Mark Smyth, Philippe Bouillet

**Affiliations:** 1 The Walter and Eliza Hall Institute of Medical Research, 1G Royal Parade, Parkville, Victoria 3050, Australia; 2 The Peter MacCallum Cancer Centre, St Andrews Place, East Melbourne, Victoria 3000, Australia

## 

The FasL/Fas system is critical for deletion of autoreactive and antigen-activated T and B cells. Accordingly, mutations in these proteins result in lymphadenopathy and autoimmunity in *gld *and *lpr *mutant mice, which lack functional FasL or Fas, respectively. Upon antigenic stimulation of T cells, FasL is sythesised, directed to and stored in secretory lysosomes followed by extrusion at the immunological synapse where it is rapidly downregulated by a metalloprotease, shedding the extracellular portion (sFasL) to prevent non-specific killing. It is unclear whether the pathology observed in *gld *mutant mice is due to the loss of the membrane-bound or the secreted form of FasL or both.

We have produced a panel of mutant FasL knock-in mice to address this question. In the first mutant strain the cytoplasmic and trans-membrane domains of FasL were replaced with the signal peptide from G-CSF. Activated T cells from these mutant mice can produce cytoplasmic but no membrane bound FasL and, interestingly, they are defective in FasL-mediated cytotoxic function and undergo significantly less activation-induced cell death upon re-stimulation with anti-CD3 antibodies than wt T cells. The extent of these defects is similar to that seen in FasL mutant *gld *T cells. With age these FasL mutant knock-in mice develop lymphadenopathy and splenomegaly and CD3^+^B220^+^CD4^-^CD8^- ^T cells accumulate, similarly to what has been observed in *gld *and *lpr *mutant mice. In contrast to *gld *mice, the FasL mutant knock-in mice on the C57BL/6 background develop haemopoietic tumours and reticular cell sarcomas, suggesting that while membrane-bound FasL is the guardian against autoimmunity, secreted FasL may play a critical role in tissue damage and tumour suppression.
